# Supplementary material for: Organic photovoltaic mini-module providing more than 5000 V for energy autonomy of dielectric elastomer actuators
Source: Nat Commun. 2025 Feb 28;16:2048. doi: 10.1038/s41467-025-57226-6 (PMC11871061; doi:10.1038/s41467-025-57226-6)
Supplement: Supplementary file 1 — Supplementary Information [file 41467_2025_57226_MOESM1_ESM.pdf]

Supplementary Information

Organic photovoltaic mini-module providing more than 5000 volts for energy autonomy of dielectric elastomer actuators

Ershuai Jiang[a,b†], *Armin Jamali*[a, c†], *Mathias List*[b,d], *Dushyant Bhagwan Mishra*[a,c], *Seyed Alireza Sheikholeslami*[a,c], *Frank Goldschmidtboeing*[a,c], *Peter Woias*[a,c], *Clemens Baretzky*[b,d], *Oliver Fischer*[b,f], *Lasse Bienkowski*[b], *Birger Zimmermann*[b], *Stefan W. Glunz*[a,b,f], *Uli Würfel*[a,b,d]*

[a]Cluster of Excellence livMatS, University of Freiburg, Georges-Köhler-Allee 105, 79110 Freiburg, Germany

[b]Fraunhofer Institute for Solar Energy Systems ISE, Heidenhofstr. 2, 79110 Freiburg, Germany

[c]Faculty of Engineering, Department of Microsystems Engineering (IMTEK), University of Freiburg, Georges-Köhler-Allee 101, 79110 Freiburg, Germany

[d]Freiburg Materials Research Center FMF, University of Freiburg, Stefan-Meier-Str. 21, 79104 Freiburg, Germany

[f]Chair of Phototovoltaic Energy Conversion, Department of Sustainable Systems Engineering INATECH, University of Freiburg, Emmy-Noether-Str. 2, 79110 Freiburg, Germany

[†]*These authors contributed equally to this work.*

E-mail: uli.wuerfel@ise.fraunhofer.de

Contents

Supplementary Methods 4

Supplementary Notes 7

Supplementary Note 1. Calculation of *PCE* under AM 1.5G for HV-OPMs 7

Supplementary Note 2. Breakdown of solar cells and partial shading of solar modules 7

Supplementary Note 3. DEA suction cup working principle 9

Supplementary Note 4. Equivalent circuit of solar modules 10

Supplementary Note 5. DEA suction cup capacitance 10

Supplementary Note 6. Gripping and non-gripping models 11

Supplementary Note 7. Viscoelastic behavior of dielectric elastomer actuators 12

Supplementary Figures 14

Supplementary Fig. 1 | GFF as a function of P4 line width and sub-cell width 14

Supplementary Fig. 2 | Shunt robustness of different electron transport layers 15

Supplementary Fig. 3 | Optimization of electron transport layer for PM6:GS-ISO solar cells 16

Supplementary Fig. 4 | DC breakdown strength of the encapsulation material through P4 line. 17

Supplementary Fig. 5 | Microscopic images of laser structured single solar cells. 18

Supplementary Fig. 6 | Air exposure test for PV-X plus solar cells. 19

Supplementary Fig. 7 | Air exposure test for PM6:GS-ISO solar cells 20

Supplementary Fig. 8 | Extrapolation of V_{OC} and *FF* 21

Supplementary Fig. 9 | Possible conductive residues led shunts in P4 lines 22

Supplementary Fig. 10 | Microscopic and electroluminescence images of aged 2-row modules 23

Supplementary Fig. 11 | Irreversible breakdown of solar cells under reverse bias 24

Supplementary Fig. 12 | Fitting of current-voltage curves of solar cells under reverse bias 25

Supplementary Fig. 13 | Calculated current-voltage curves of shaded and non-shaded sub-cells 26

Supplementary Fig. 14 | Risk parameters as a function of the number of shaded sub-cells 27

Supplementary Fig. 15 | Impact of large reverse bias on the performance of solar cells 28

Supplementary Fig. 16 | Fabrication process of dielectric elastomer suction cup 29

Supplementary Fig. 17 | Photograph of a fully soft dielectric elastomer suction cup 30

Supplementary Fig. 18 | Deformation of a dielectric elastomer actuator under voltage 31

Supplementary Fig. 19 | Transient suction pressure generated by the suction cup. 32

Supplementary Fig. 20 | Equivalent circuit of solar cell. 33

Supplementary Fig. 21 | Charging current of the dielectric elastomer actuator suction cup 34

Supplementary Fig. 22 | Maximum power (P_{max}) of the PM6:GS-ISO photovoltaic mini-module .. 35
Supplementary Fig. 23 | Viscoelastic behavior of the dielectric elastomer actuator. 36
Supplementary Fig. 24 | Details energy analysis 37
Supplementary Fig. 25 | Turning on feature of the warm white LED lamp..................................... 38
Supplementary Tables 39
Supplementary Table 1. Photovoltaic performance of high-voltage photovoltaic mini-modules. ... 39
Supplementary Table 2. Photovoltaic performance of single solar cells 40
Supplementary Table 3. Irreversible breakdown voltage (V_{br}), current density (J_{br}) and power density (P_{br}) of solar cells. 40
Supplementary Table 4. The parameters of PM6:GS-ISO mini-module used for modeling 41
Supplementary Table 5. The parameters of the DEA suction cup used for modeling 41
References 42

Supplementary Methods

The materials used for the devices presented in the Supplementary Methods are identical with those used in the main text. For the experiments related to the optimization of electron transport layers, air exposure test, potential induced degradation test and breakdown test, small area solar cells were built on 25 × 25 mm^2 substrates with pre-structured Indium Tin Oxide (ITO). The active area of 0.0925 cm^2 was defined by thermally evaporated top electrodes using a shadow mask. These solar cells are called C-type solar cells in the following and they were fabricated based on the same parameters of spin coating speeds, annealing conditions, etc. as those for the high-voltage organic photovoltaic mini-modules (HV-OPMs) in the main text.

Shunt robustness of electron and hole transport layers

In organic solar cells processed from solution, it can happen that the photoactive layer comprises a pinhole, e.g., by coating defects. This then leads to a direct contact between the electron transport layer (ETL) and hole transport layer (HTL), i.e., a shunt. As the mentioned pinholes of the photoactive layer cannot be completely avoided, it is important that the formed junction between ETL and HTL provides a certain resistance to the flow of charge carriers as this constitutes a loss in an operating solar cell. To test the shunt-proof abilities of different ETLs, ZnO, PFN-Br, and ZnO/PFN-Br layers were spin coated on pre-structured ITO glass, with a speed of 2000 rpm for ZnO and 4000 rpm for PFN-Br. The ZnO layer was annealed under 130 °C for 10 min, followed by HTL-1 spin coating. After that, 5 nm Au and 70 nm Ag were thermally evaporated with a shadow mask to form an active area of 0.0925 cm^2. Finally, the current density-voltage (*JV*) curves were measured with a source-measurement unit (SMU) Keithley 2400. The contact resistances were calculated as the reciprocals of the slopes of the *JV* curves near $V = 0\,\mathrm{V}$. As can be seen in Supplementary Fig. 2, the use of ZnO improves the shunt proofness by almost 3 orders of magnitude. The combination of ZnO and PFN-Br shows the largest resistance values.

Optimization of electron transport layer for PM6:GS-ISO devices

To optimize the electron transport layer for PM6:GS-ISO devices, C-type solar cells were fabricated. Different ETLs of PFN-Br, ZnO and ZnO/PFN-Br were spin coated, with a speed of 2000 rpm for ZnO and 4000 rpm for PFN-Br. The ZnO layer was annealed at 130 °C for 10 min. Then, PM6:GS-ISO layer was spin coated with a speed of 2500 rpm and annealed at 160 °C for 10 min. After it, a layer of HTL-1 was spin coated with a speed of 4000 rpm,

followed by Au/Ag deposition. The *JV* curves of the solar cells were measured under 10 klux warm white LED illumination, with a SMU (Keithley 2400).

Air exposure test

To determine the effect of air exposure for different layers on the performance of the devices, C-type solar cells were fabricated. The coating procedures of ZnO, ZnO/PFN-Br and HTL-1 were the same as the ones used for the HV-OPMs. The 'Before Au/Ag' samples were exposed to air for 30 min before Au/Ag deposition. The 'After Au/Ag' samples were exposed to air for 30 min after Au/Ag deposition. The 'Without' samples were not exposed to air. The *JV* curves of the solar cells were measured under 10 klux warm white LED illumination, with a SMU Keithley 2400.

Electrical breakdown test of encapsulant for P4 lines

To measure the electrical breakdown voltage of the encapsulant for the P4 line, a full layer stack identical to the PM6:GS-ISO modules were fabricated, and a structure line was laser patterned with the same parameters used for the P4 lines. The encapsulation process was identical to the one used for the HV-OPMs. Then, a forward voltage sweep was provided by a high voltage amplifier (10HVA24-P1) and the current was recorded.

Electroluminescence imaging of aged 2-row modules

The setup used to measure electroluminescence (EL) images of aged 2-row modules was the same as the one used for the HV-OPMs in the main text, but the voltage was supplied by the SMU Keithley 2636A directly. For PM6:GS-ISO based 2-row modules, a forward bias of 140 V was used. 100 V and 150 V forward biases were used for PV-X plus 2-row module aged under short circuit and open circuit conditions, respectively. In general, the voltages were selected as low as possible to guarantee a good signal to noise ratio.

Measuring viscoelastic behaviour of the dielectric elastomer actuator

To measure the viscoelastic behaviour of the dielectric elastomer actuator (DEA), a disk-shaped specimen with a diameter of 28 mm and a thickness of 2 mm, made of Ecoflex 00-10 mixed with the silicone thinner (the same as the elastomer of the DEA suction cup) was fabricated with molding techniques. To perform a creep test, a universal testing machine (Inspekt table, Hegewald & Peschke, Germany) was used to apply a controlled compressive force (0.95 N) to the specimen and log the strain values for one hour. This force led to a

compressive stress of 1.54 kPa on the specimen, which is equal to the amount of stress exerted on the DEA suction cup when subjected to approximately 5 kV from the HV-OPM under 100 klux. A generalized Maxwell model with a single spring element in parallel with 3 spring-dashpot lines was considered for modelling the viscoelastic behaviour of the elastomer.

Supplementary Notes

Supplementary Note 1. Calculation of *PCE* under AM 1.5G for HV-OPMs

The I_{SC} of the HV-OPV based on PV-X plus was calculated by integrating EQE with the AM 1.5G spectrum:

$$I_{\mathrm{SC,AM1.5G}} = A_{\mathrm{sub-cell}} \int_0^{\infty} \mathrm{q} EQE(\lambda) S(\lambda) \mathrm{d}\lambda \quad (1)$$

where $A_{sub\text{-}cell}$ is the area of a sub-cell, q the elementary charge and S(λ) the irradiance spectrum of AM 1.5G. This gives a result of I_{SC} = 60.7 µA. The mismatch ratio of LED lamp 100 klux and AM 1.5G was calculated as:

$$R_{\mathrm{mis}} = \frac{I_{\mathrm{SC,AM1.5G}}}{I_{\mathrm{SC,LED\ 100\ klux}}} \quad (2)$$

This gives a result of R_{mis}=2.14, which means that the AM 1.5G equals to 214 klux for PV-X plus based HV-OPMs. To be closer to real conditions, we use the average value of the measured $I_{SC,LED\ 100\ klux}$ = 28.9 µA here.

The V_{OC} under AM 1.5G was extrapolated from the log-linear plot of illuminance (I_{il}) dependent V_{OC} by a linear fitting, as illustrated in Supplementary Fig. 8a. With this, the V_{OC} was calculated to be 3875 V under AM 1.5G.

The *FF* under AM 1.5G was extrapolated from the linear plot of illuminance dependent FF by a linear fitting, as illustrated in Supplementary Fig. 8b. The result was 0.565.

The *PCE* under AM 1.5G was then calculated as usual by:

$$PCE = \frac{I_{\mathrm{SC,AM1.5G}} V_{\mathrm{OC,AM1.5G}} FF_{\mathrm{AM1.5G}}}{A_{\mathrm{active}} P_{\mathrm{in}}} \quad (3)$$

where A_{active}=13.7 cm^2 is the active area of the HV-OPM and P_{in}=100 mW/cm^2 is the power intensity of the AM 1.5G. With these numbers, the *PCE* under AM 1.5G results in $PCE_{AM1.5G}$ = 10.2%.

Supplementary Note 2. Breakdown of solar cells and partial shading of solar modules

When a solar cell is under strong reverse bias, there is a risk of breakdown. Generally, there are three types of breakdown that can occur for solar cells under negative bias, namely tunneling breakdown, Avalanche breakdown and thermal instability[1]. For OPV at room temperature, the thermal instability could be the main reason for irrevesible breakdown, while the tunneling current is dominant under high reverse bias voltage[1]. To describe tunneling

current, Poole-Frenkel model and Fowler-Nordheim model are commonly used[2]. Compared to the Poole-Frenkel tunneling, the Fowler-Nordheim tunneling occurs at a higher bias voltage, which is descibed as:

$$J_{\mathrm{FN}} = \frac{\mathrm{mq}^3 E^2}{8\mathrm{m}^* \pi \mathrm{h} \phi_{\mathrm{FN}}} \exp\left(\frac{-8\pi\sqrt{2\frac{\mathrm{m}^*}{\mathrm{m}}\phi_{\mathrm{FN}}^3}}{3\mathrm{qh}E} \right) \tag{4}$$

Where m and m* are electron mass and effective electron mass, q the elementary charge, E the electric field strength, h the Planck constant, and ϕ_{FN} the potential barrier.

To study the breakdown risk of our HV-OPMs, we measured the *IV* curves of single solar cells under revese bias. As shown in Supplementary Fig.11 and Supplementary Table 3, the breakdown voltages (V_{br}) were rather high for PV-X plus and PM6:GS-ISO solar cells. To obtain parameters to describe the reverse current, we fitted the measured curves with:

$$J_{\mathrm{FN}} = \mathrm{a}V^2 \exp\left(\frac{b}{V}\right) \tag{5}$$

As shown in Supplementary Fig. 12, the parameters (a,b) resulted to (-749.1, 394.7) and (-1.688, 166.1) for PV-X plus and PM6:GS-ISO, respectively.

When a solar module (in which the sub-cells are interconnected in series) is partially shaded, the shaded sub-cells will be under reverse bias driven by the non-shaded sub-cells. Different to breakdown test with a sourcemeter (that can provide both a large voltage and a large current), the voltage (V_{sha}) and injected current (I_{sha}) applied to the shaded sub-cells are limited by the non-shaded part. To determine the V_{sha} and I_{sha}, we flipped the *IV* curves of the shaded part and the non-shaded part into the first quadrant (Supplementary Fig. 13). The I_{sha} was calculated as:

$$I_{sha} = -A_{\mathrm{sub-cell}} \frac{\mathrm{a}}{N_{\mathrm{sha}}^2} V^2 \exp\left(-\frac{\mathrm{b}N_{\mathrm{sha}}}{V}\right) \tag{6}$$

where N_{sha} is the number of shaded sub-cells and $A_{\mathrm{sub-cell}}$ the area of sub-cells. The *IV* curves of the series connection of the working sub-cells were determined by:

$$I_{\mathrm{non-sha}} = \frac{A_{\mathrm{sub-cell}}}{A_{\mathrm{single-cell}}} \left(I_{\mathrm{ph}} - I_0 \left(\exp\left(\frac{V_{\mathrm{non-sha}} + I_{\mathrm{non-sha}}(5024-N_{\mathrm{sha}})R_{\mathrm{s}}}{(5024-N_{\mathrm{sha}})n_{\mathrm{id}}V_{\mathrm{th}}}\right) - 1\right) - \frac{V_{\mathrm{non-sha}} + I_{\mathrm{non-sha}}(5024-N_{\mathrm{sha}})R_{\mathrm{s}}}{(5024-N_{\mathrm{sha}})R_{\mathrm{p}}}\right) \tag{7}$$

where I_{ph}, I_0, R_{s}, R_{p} and n_{id} are parameters derived from the single solar cells (Supplementary Fig. 5). The derivation process can be seen in Supplementary Note 4. The V_{sha} was determined as the crossing point of *IV* curves for shaded and non-shaded parts. From the Supplementary

Fig. 13 it can be seen that the operating point of the non-shaded sub-cells approaches open circuit conditions when N_{sha} is ≥ 300 for PM6:GS-ISO and ≥ 150 for PV-X plus. As a consequence, the I_{sha} is strongly limited. On the contrary, when N_{sha} is small, the I_{sha} is close to I_{SC} of the working sub-cells, but the V_{sha} is rather low. Supplementary Fig. 14 shows the reverse bias voltage per sub-cell and the power density dissipated in the shaded sub-cells, respectively. It can be seen that the less shaded sub-cells, the higher the risk of breakdown. To quantify this risk, we define a breakdown index (*BRI*) by:

$$BRI = \frac{I_{\mathrm{sha}}V_{\mathrm{sha}} - N_{\mathrm{sha}}A_{\mathrm{sub-cell}}P_{\mathrm{br}}}{N_{\mathrm{sha}}A_{\mathrm{sub-cell}}P_{\mathrm{br}}} \quad (8)$$

When the *BRI* is negative, it means that the power dissipated in the shaded sub-cells is lower than that leading to an irreversible breakdown, thus the shaded sub-cells will not be damaged.

In addition, we applied a high reverse bias smaller than the V_{br} to the C-type solar cells.The results show clearly that no performance degradation is observed for reverse voltages of 35 V for PV-X plus and 30 V for PM6:GS-ISO, respectively. Instead, the reverse bias even led to a slight improvement of the fill factor (Supplementary Fig. 15). This is most probable due to shunt-burning (of minor shunts) but it is beyond the scope of this work to analyze this in detail. The important result here is the remarkable stability against high reverse bias.

Supplementary Note 3. DEA suction cup working principle

DEAs are made of soft and thin non-conductive elastomer layers sandwiched between two compliant electrode layers. When a DEA is subjected to a high electric field, the Coulomb forces between the opposite charges accumulated on the electrodes induce a mechanical stress onto the elastomer that is called Maxwell stress. Due to this Maxwell stress, the elastomer is compressed in the direction perpendicular to the electrodes and also expands in area. The area expansion occurs mainly due to the fact that the Poisson's ratio of the elastomer is close to 0.5, which makes them rather incompressible. For the DEA suction cup in this work, the dome expands due to Maxwell stress when a high voltage is applied to the electrodes. When the suction cup establishes an airtight seal against the surface of an object, the subsequent expansion of the dome's internal volume generates a localized pressure differential. This pressure differential, with the external pressure exceeding the internal pressure of the suction cup, results in the object being grasped and held securely in place by the suction force.

Supplementary Note 4. Equivalent circuit of solar modules

In Supplementary Fig. 20, the single diode model with a parallel capacitor was used to model a single solar cell. The *IV* relationship of a single solar cell can be written as:

$$I(V) = -I_{\mathrm{ph}} + I_{\mathrm{d}} + I_{\mathrm{p}} + I_{\mathrm{c}} \tag{9}$$

$$I(V) = -I_{\mathrm{ph}} + I_0\left(\exp\left(\frac{V-IR_{\mathrm{s}}}{n_{\mathrm{id}}V_{\mathrm{th}}}\right) - 1\right) + \frac{V-IR_{\mathrm{s}}}{R_{\mathrm{p}}} + C_{\mathrm{p}}\frac{\mathrm{d}(V-IR_{\mathrm{s}})}{\mathrm{d}t} \tag{10}$$

where I_{ph} is the photocurrent, I_0 the reverse saturation current, R_{s} the series resistance, R_{p} the parallel resistance, n_{id} the ideality factor, C_{p} the parallel capacitance and $V_{\mathrm{th}} = \mathrm{k}T/q$ the thermal energy, with q the elementary charge, k the Boltzmann constant and *T* the temperature. Considering a solar module including *N* sub-cells and assuming every sub-cell to work in the very same manner, the voltage and current of the module are given by:

$$V_{\mathrm{m}} = V_1 + V_2 + \cdots V_{\mathrm{N-1}} + V_{\mathrm{N}} = \mathrm{N}V_{\mathrm{i}} \tag{11}$$

$$I_{\mathrm{m}} = I_1 = I_2 \ldots. = I_{\mathrm{N}} = I_{\mathrm{i}} \tag{12}$$

Where V_{m} and I_{m} are voltage and current of the module, V_{i} and I_{i} the voltage and current of the sub-cell with number i. Combining Equations (10-12), we get:

$$I_{\mathrm{m}} = -I_{\mathrm{ph}} + I_0\left(\exp\left(\frac{V_{\mathrm{m}}-I_{\mathrm{m}}\mathrm{N}R_{\mathrm{s}}}{\mathrm{N}n_{\mathrm{id}}V_{\mathrm{th}}}\right) - 1\right) + \frac{V_{\mathrm{m}}-I_{\mathrm{m}}\mathrm{N}R_{\mathrm{s}}}{\mathrm{N}R_{\mathrm{p}}} + \frac{C_{\mathrm{p}}}{\mathrm{N}}\frac{\mathrm{d}(V_{\mathrm{m}}-I_{\mathrm{m}}\mathrm{N}R_{\mathrm{s}})}{\mathrm{d}t} \tag{13}$$

Comparing Equations (10) and (7), one can deduce the parameters for the module:

$R_{\mathrm{s,m}}=NR_{\mathrm{s}}$, $R_{\mathrm{p,m}}=NR_{\mathrm{p}}$, $n_{\mathrm{id,m}}=Nn_{\mathrm{id}}$, $C_{\mathrm{p,m}}=C_{\mathrm{p}}/N$.

The geometrical capacitance of a single cell C_{p} is measured to 66 pF in this work. The total number of sub-cells is $N = 5024$ and thus very large. As a result, $C_{\mathrm{p,m}}$=13.1 fF, which can safely be neglected.

The parameters used for modelling were fitted with measured *IV* curves and are listed in Supplementary Table 4.

Supplementary Note 5. DEA suction cup capacitance

We derive this model for the case of an unloaded (i.e. non-gripping) suction cup. In this case the DEA is virtually free to move and no external force by the produced suction pressure has

to be considered. We start with the equation for the capacitance depending on geometric parameters. DEA is basically a set of n plate capacitors connected in parallel, with the area *A*, distance *d* and a dielectric with a dielectric permittivity of $\varepsilon_\mathrm{r}\varepsilon_0$. Its total capacitance is therefore given by:

$$C_\mathrm{DEA} = n\varepsilon_\mathrm{r}\varepsilon_0 \cdot \frac{A}{d} \tag{14}$$

We define the ratio of plate distance *d* and initial unstrained distance d_0 as the stretch ratio λ:

$$\lambda = \frac{d}{d_0} \tag{15}$$

The DEA is made of silicone, that can be considered as incompressible, i.e., the product of area *A* and distance *d* is constant. This argument helps to express the capacitance in terms of the initial unstrained values A_0 and d_0 and the stretch ratio as:

$$C_\mathrm{DEA} = n\varepsilon_r\varepsilon_0 \frac{A_0}{d_0}\lambda^2 = C_\mathrm{DEA,0} \cdot \lambda^2 \tag{16}$$

For the sake of simplicity, the elastic behavior of the silicone can be approximated by Hooke's law with an elastic modulus *Y*:

$$S = 1 - \lambda = \frac{T_\mathrm{maxwell}}{Y} \tag{17}$$

with the engineering strain *S* and the Maxwell stress according to:

$$T_\mathrm{Maxwell} = \frac{1}{2}\varepsilon_\mathrm{r}\varepsilon_0 \frac{V_\mathrm{DEA}^2}{d} \tag{18}$$

The final implicit equation for the capacitance C_DEA is derived by combining equations (14) to (18) as:

$$\frac{C_\mathrm{DEA,0}}{C_\mathrm{DEA}}\left(1 - \sqrt{\frac{C_\mathrm{DEA,0}}{C_\mathrm{DEA}}}\right) = \frac{1}{2}\frac{\varepsilon_\mathrm{r}\varepsilon_0}{Yz_0^2}V_\mathrm{DEA}^2 \tag{19}$$

Therein, z_0 is the initial DEA layer thickness. This approach was already validated in reference[3].

Supplementary Note 6. Gripping and non-gripping models

When charging up the DEA suction cup in a state of non-gripping, the DEA is free deforming without external force due to pressure difference between inside and outside of the suction cup. Furthermore, a free-standing DEA suction cup is confined even less without the limitation at

the edge of the dome. Thus, it can be imagined that the charging current of different states are different. Supplementary Fig. 21 shows the charging currents under 100 klux for the experimental gripping and non-gripping states, as well as the simulated free-standing state of the suction cup. It is visible that the simulated free state shows the highest current, the "non-gripping" curve shows a slightly lower current and the "gripping" curve shows the lowest current, although they are all very close together. For the simulation a free-standing actuator was assumed with no external loading or limitation. The real actuator is however confined by the frame that surrounds the actuator. Therefore, the actuator capacitance does not increase that much as that of the free-standing actuator and less charges are needed to build up the voltage. The gripping DEA is even more confined leading to a further decrease of the capacitance change and therefore the lowest current. These differences in charging current, though small for this design, can be used to monitor the gripping state of the suction cup. Please note that there are sharp peaks for experimental curves at the beginning of illumination, which are due to a short overshoot in light intensity when turning on the lamp (cf. Supplementary Fig. 25).

Supplementary Note 7. Viscoelastic behavior of dielectric elastomer actuators

Viscoelastic behaviour is a common issue for DEAs, which leads to creeping action and restricts the performance of the DEA, especially under high frequency[4]. Generally, silicone has slighter viscoelastic damping compared to other dielectric elastomers such as VHBs from 3M and polyurethane (PU)[5]. Nevertheless, we still observe viscoelastic behaviour in our DEA suction cup. To confirm it, we measured the compressive stress and strain of the DEA used for the suction cup. As shown in Supplementary Fig. 23, the suction cup reacts to the fast charging by a sudden deformation followed by a very slow creep motion into its final shape. The curve fitting results (with an R-square value of 0.9999) show two short retardation times of 0.48 s and 11.19 s, and a long retardation time of 114.5 s. This indicates that the creep phase may last up to several minutes and may take up to 20% of the total deformation for loads comparable to the suction cup case. Thus, the suction cup keeps on deforming during the whole measurement and therefore a permanent increase of the capacitance C_{DEA} occurs. The total delivered electrical energy by the HV-OPM comprises the fraction taken up by the DEA and the one dissipated in the measurement resistor (R_L), as shown in Supplementary Fig. 24. The main charging period only takes about 0.25 s. However, after that a deviation occurs between measured and simulated results due to the mentioned viscoelastic behavior.

Supplementary Figures

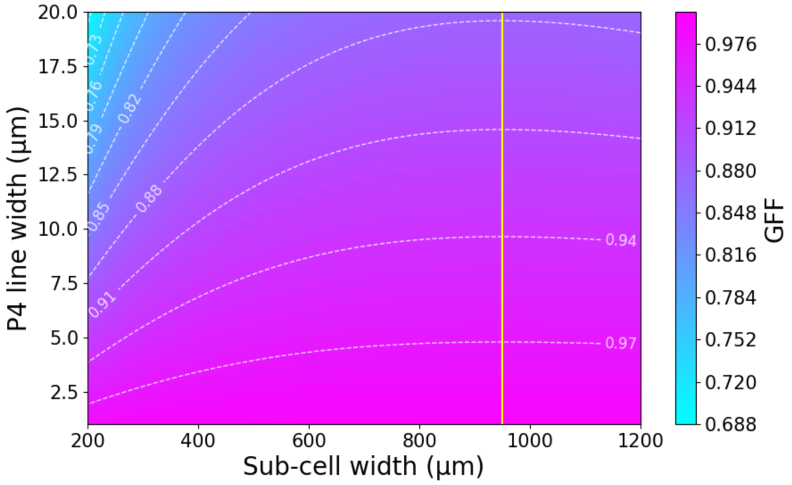

Supplementary Fig. 1 | Geometric fill factor (GFF) as a function of P4 line width and sub-cell width. The yellow line shows the maximum GFF for different widths of the P4 line.

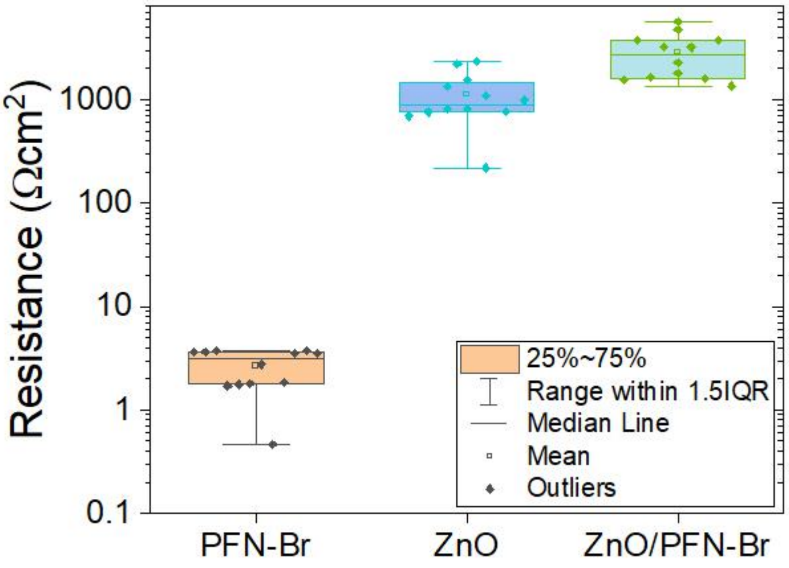

Supplementary Fig. 2 | Shunt robustness of different electron transport layers in direct contact with HTL-1.

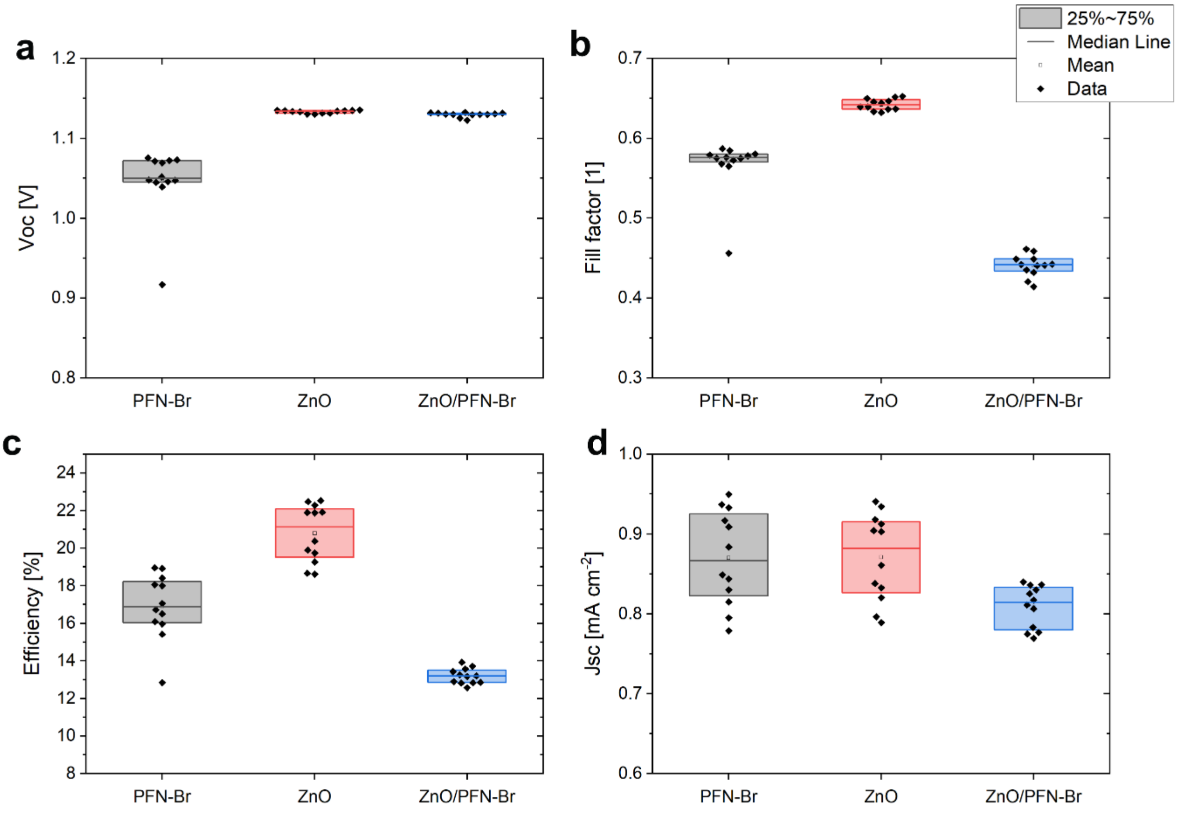

Supplementary Fig. 3 | Optimization of electron transport layer for PM6:GS-ISO solar cells. a, open circuit voltage (V_{OC}). **b**, Fill factor. **c**, Efficiency. **d**, Short circuit current density (J_{SC}). Measured under 10 klux warm white LED lamp.

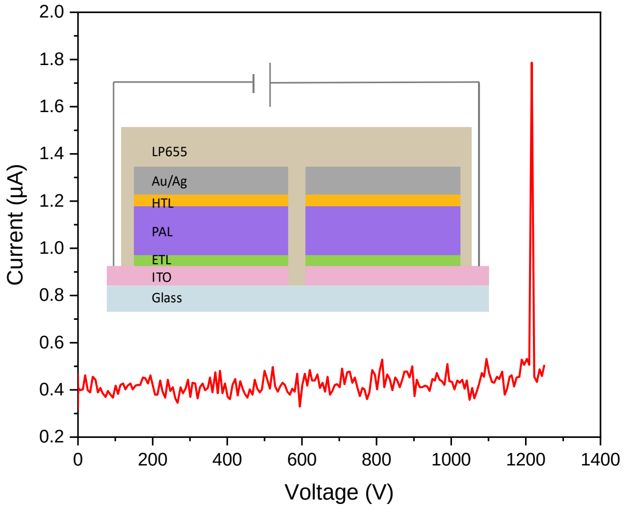

Supplementary Fig. 4 | DC breakdown strength of the encapsulation material through P4 line. A current peak occurs at 1214 V. The inset shows the architecture of the device. The gap in the middle is about 20 μm wide to mimic P4 patterns in the high voltage mini-modules, where ITO is indium tin oxide, ETL is electron transport layer, PAL is photoactive layer and HTL is hole transport layer.

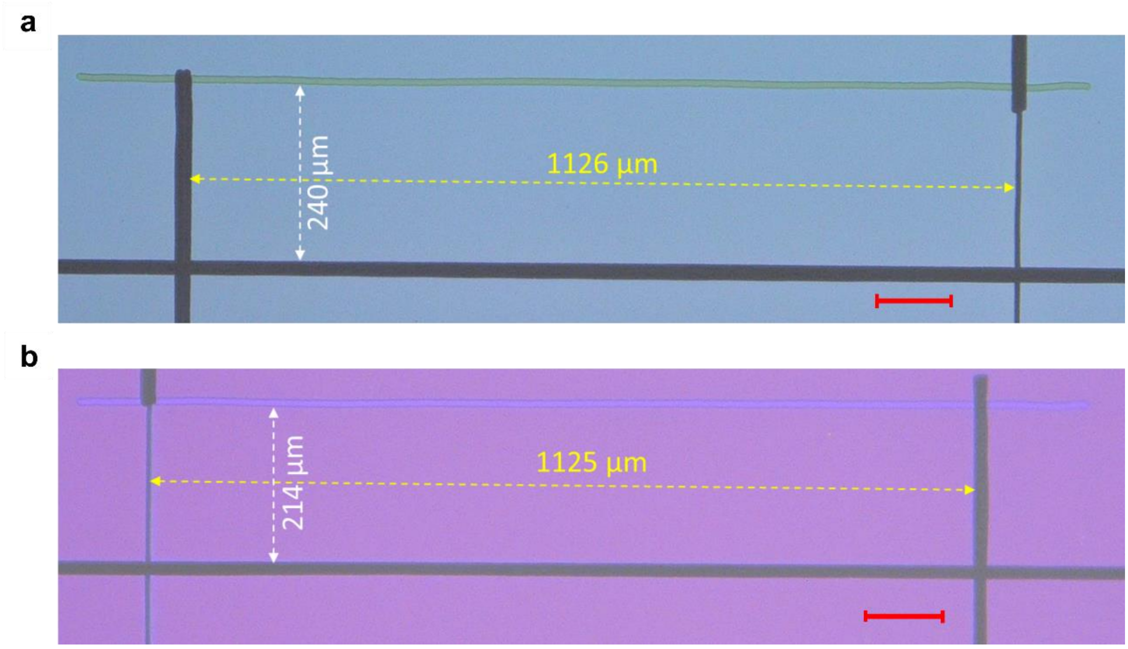

Supplementary Fig. 5 | Microscopic images of laser structured single solar cells. a, PV-X plus. **b**, PM6:GS-ISO. The scale bar is 100 µm in both cases.

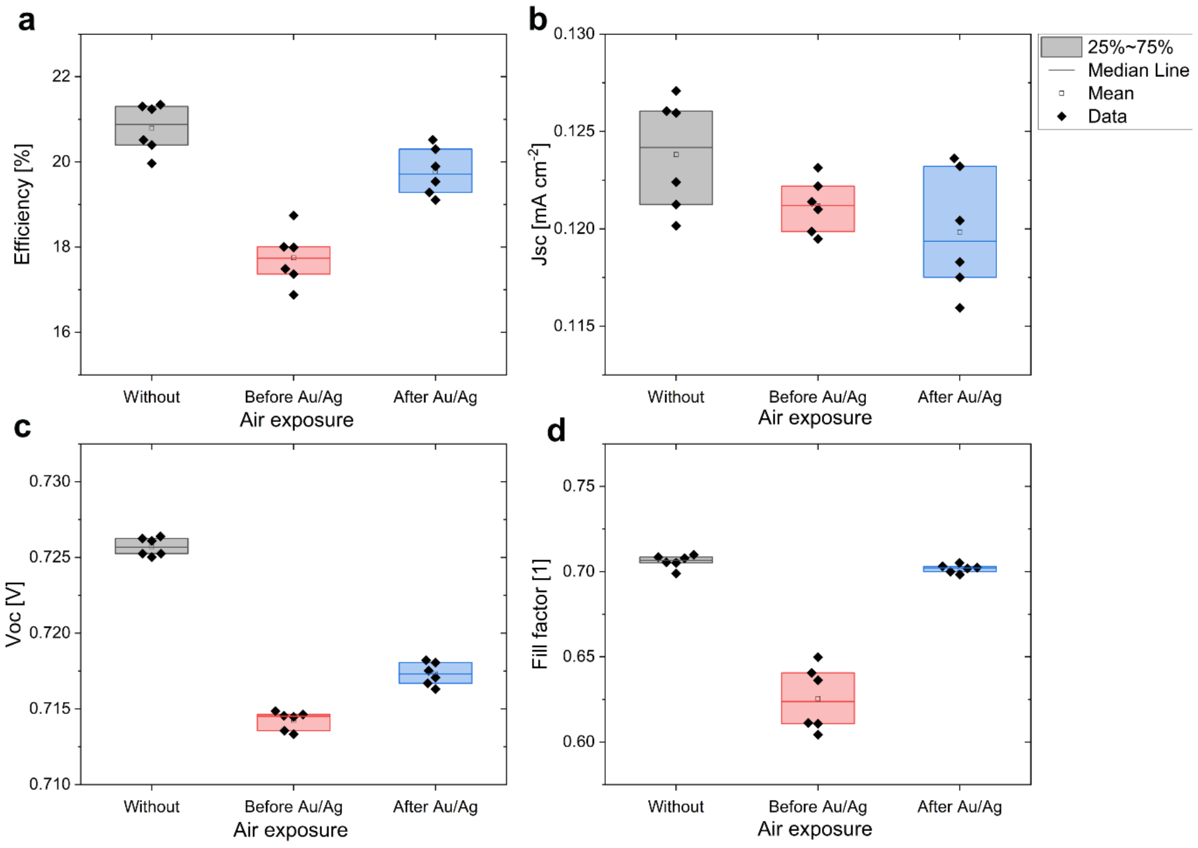

Supplementary Fig. 6 | Air exposure test for PV-X plus solar cells. a, Efficiency. **b**, Short circuit current density (J_{SC}). **c**, Open circuit voltage (V_{OC}). **d**, Fill factor. Measured under 1 klux warm white LED light.

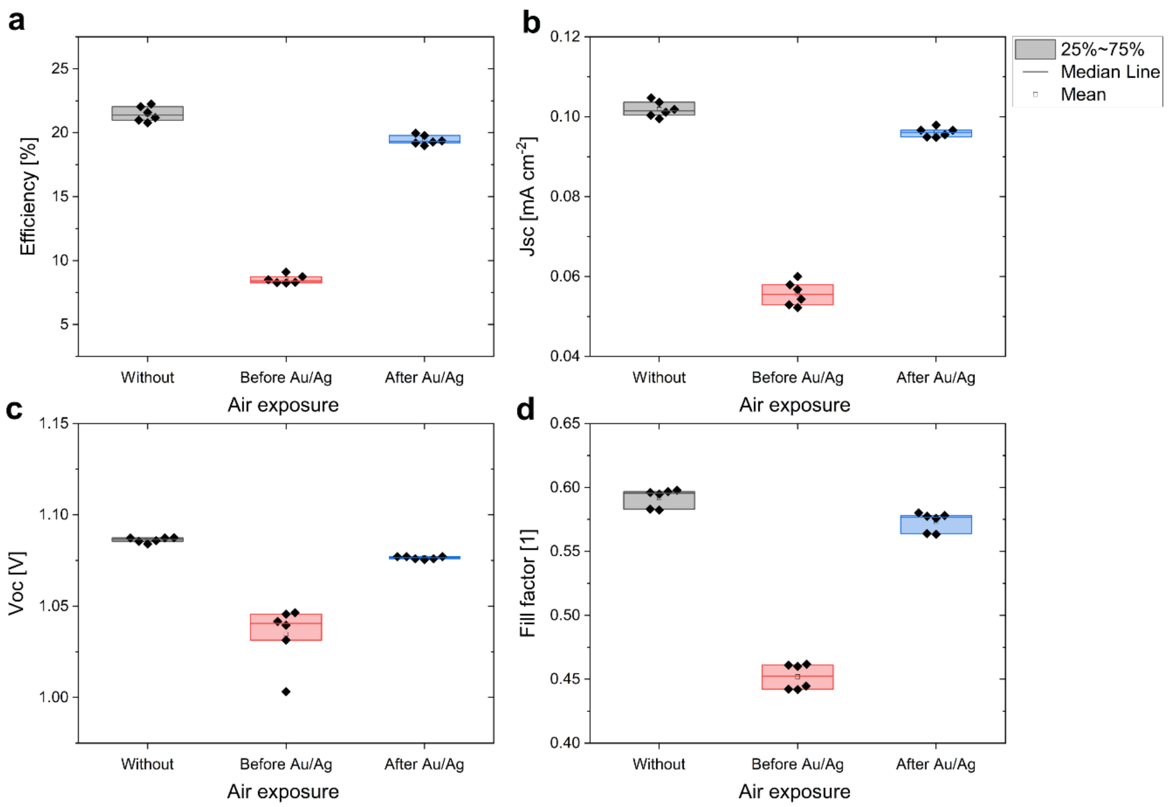

Supplementary Fig. 7 | Air exposure test for PM6:GS-ISO solar cells. **a**, Efficiency. **b**, Short circuit current density (J_{SC}). **c**, Open circuit voltage (V_{OC}). **d**, Fill factor. Measured under 1 klux warm white LED light.

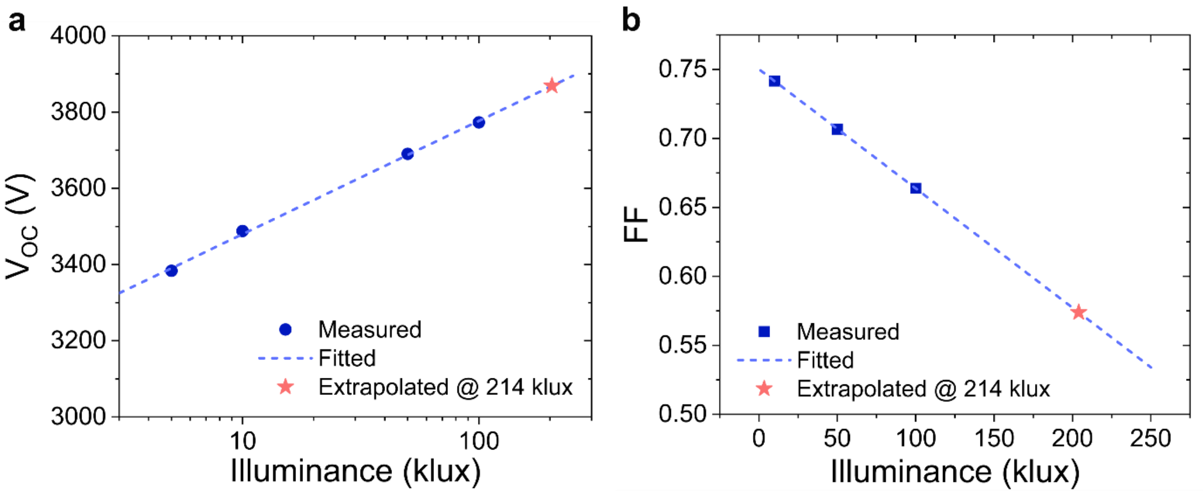

Supplementary Fig. 8 | Extraction of V_{OC} and *FF*. **a**, V_{OC}. **b**, *FF* for PV-X plus based HV-OPM.

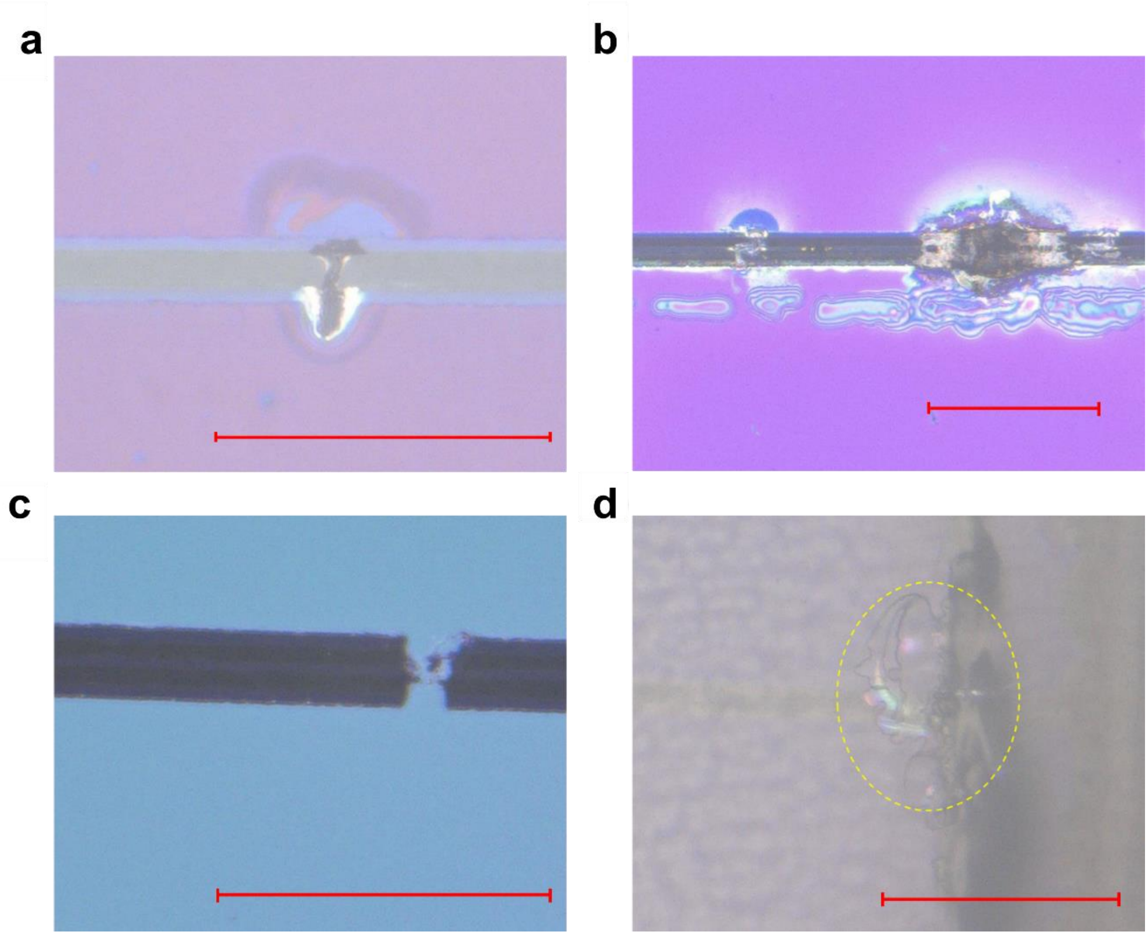

Supplementary Fig. 9 | Possible conductive residues led shunts in P4 lines. **a**, On the margin where is no metal top electrode. **b**, With full PM6:GS-ISO module layer stack. **c**. With full PV-X plus module layer stack. **d**, At the edge of the substrate where there is only ITO, the yellow dashed oval showing a potential shunt. The scalebar is 100 µm in all cases.

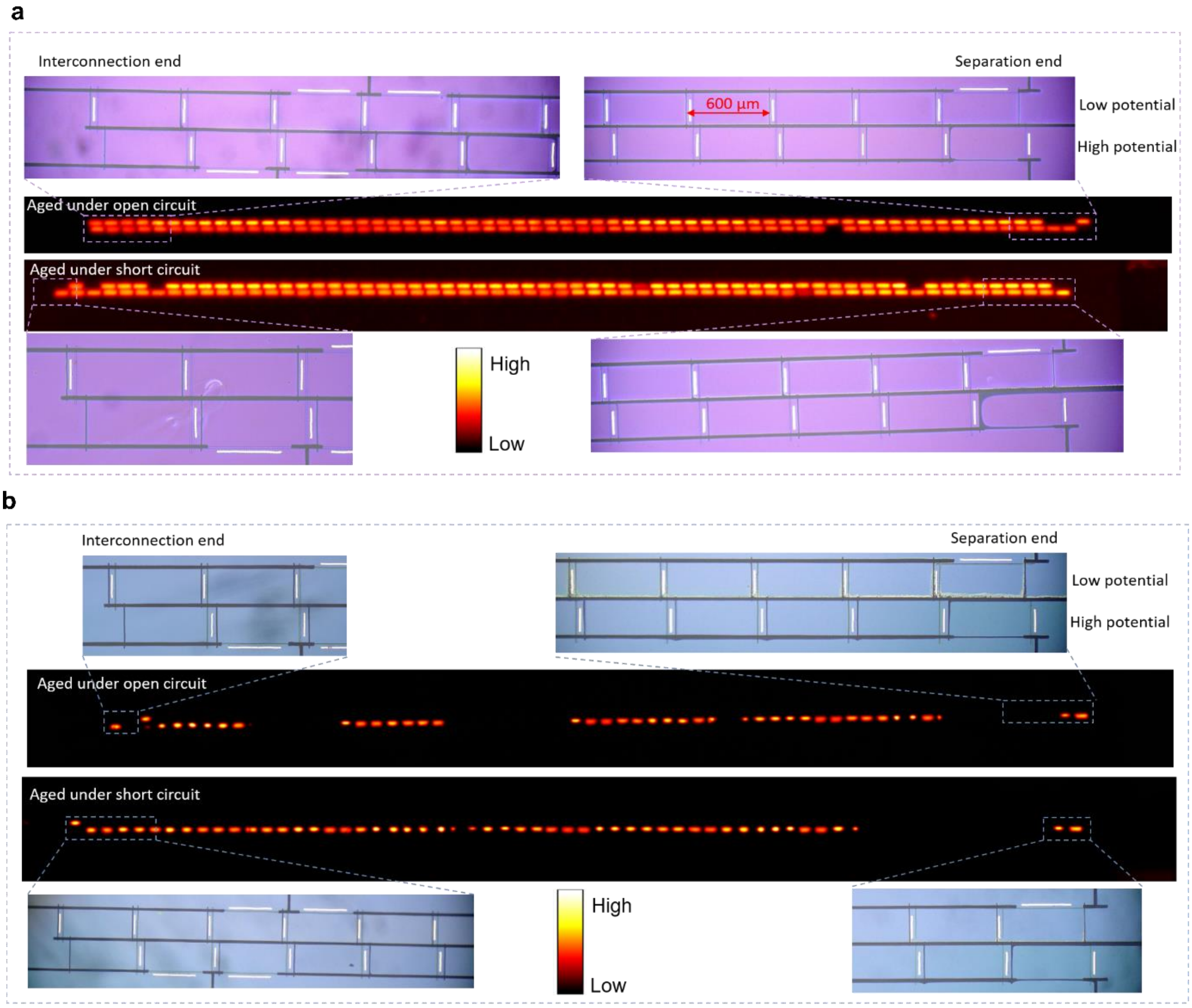

Supplementary Fig. 10 | Microscopic and electroluminescence images of aged 2-row modules. **a**, PM6:GS-ISO. **b**, PV-X plus. The modules were aged under 50 klux cool white LED light for more than 1000 hours.

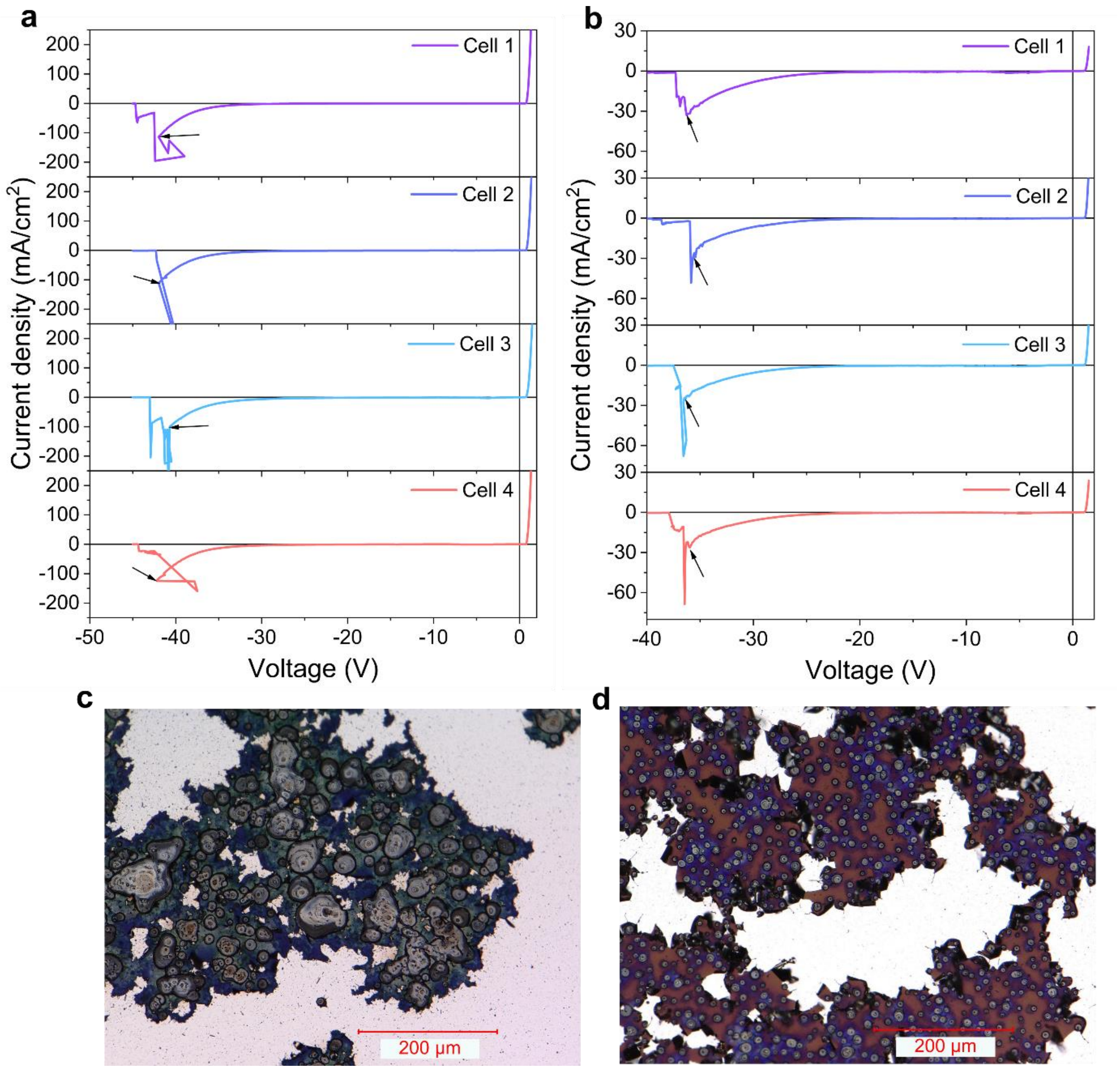

Supplementary Fig. 11 | Irreversible breakdown of solar cells under reverse bias. **a**, Current density-voltage curves of PV-X plus solar cells. **b**, Current density-voltage curves of PM6:GS-ISO solar cells. **c**. Microscopic image of a PV-X plus solar cell after breakdown. **d**, Microscopic image of a PM6:GS-ISO solar cell after breakdown. The arrows in (**a**,**b**) illustrate the critical point for irreversible breakdown.

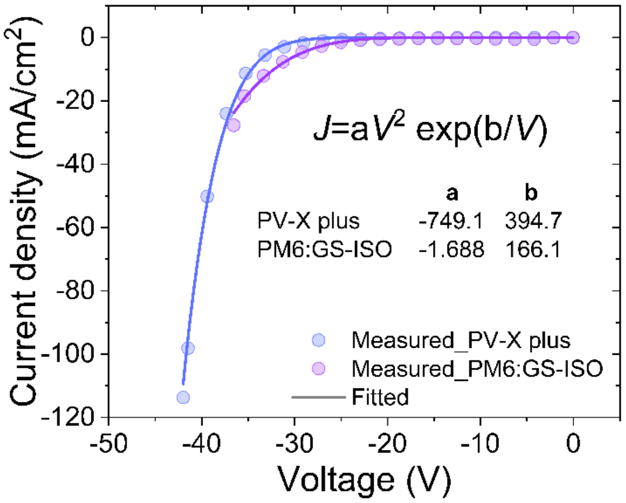

Supplementary Fig. 12 | Fitting of current-voltage curves of solar cells under reverse bias.

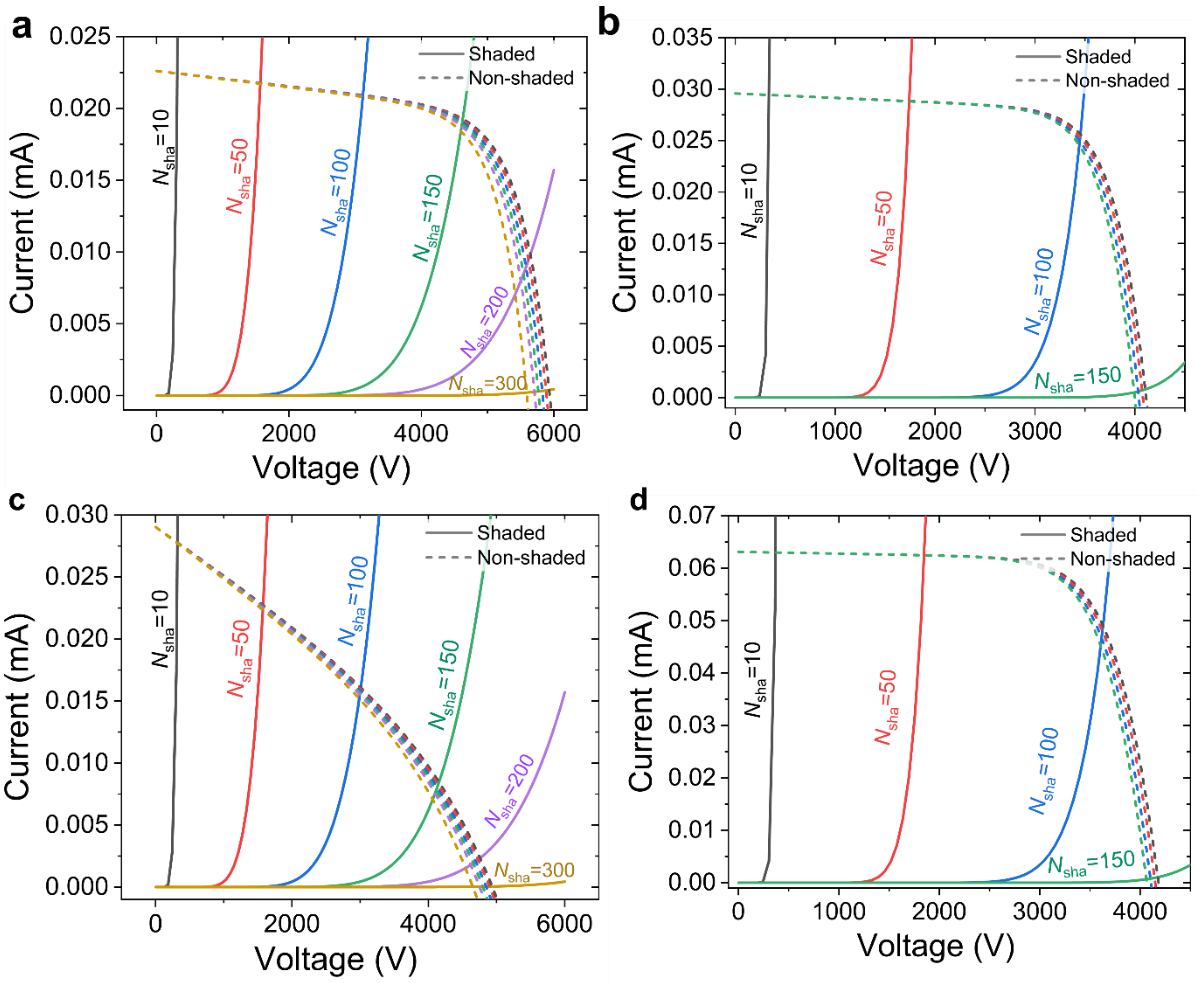

Supplementary Fig. 13 | Calculated current-voltage curves of shaded and non-shaded sub-cells. **a**, PM6:GS-ISO and **b**, PV-X plus under 100 klux LED illumination, respectively. **c**, PM6:GS-ISO and **d**, PV-X plus under AM 1.5G illumination, respectively. N_{sha} is the number of shaded sub-cells. The crossing points of the solid lines and the corresponding dashed lines were used to determine the current and voltage of the shaded sub-cells.

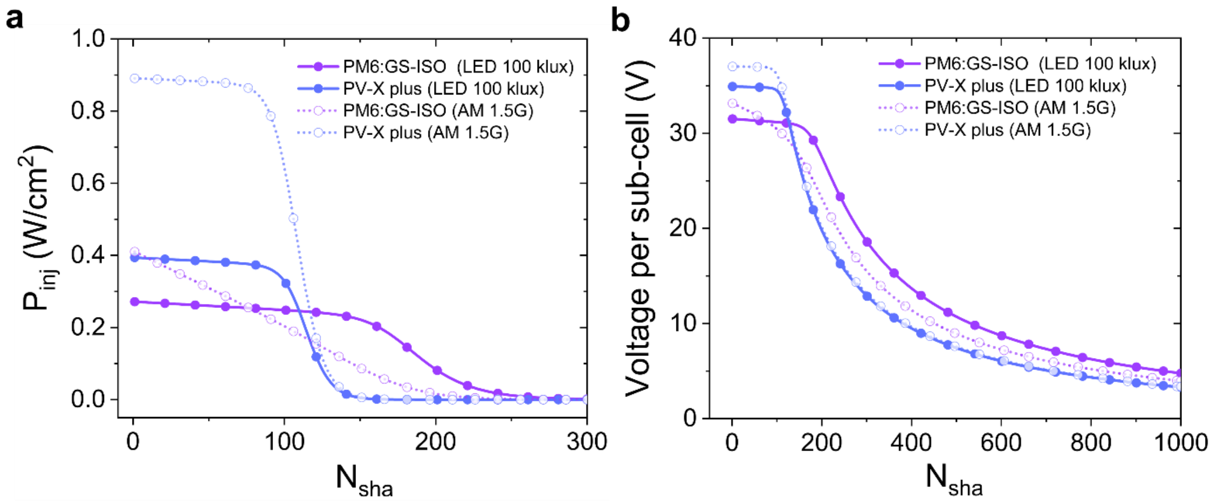

Supplementary Fig. 14 | Risk parameters as a function of the number of shaded sub-cells. **a**, Bias voltage on each shaded sub-cell. **b**, Power density applied to each shaded sub-cell (P_{inj}) by the non-shaded sub-cells.

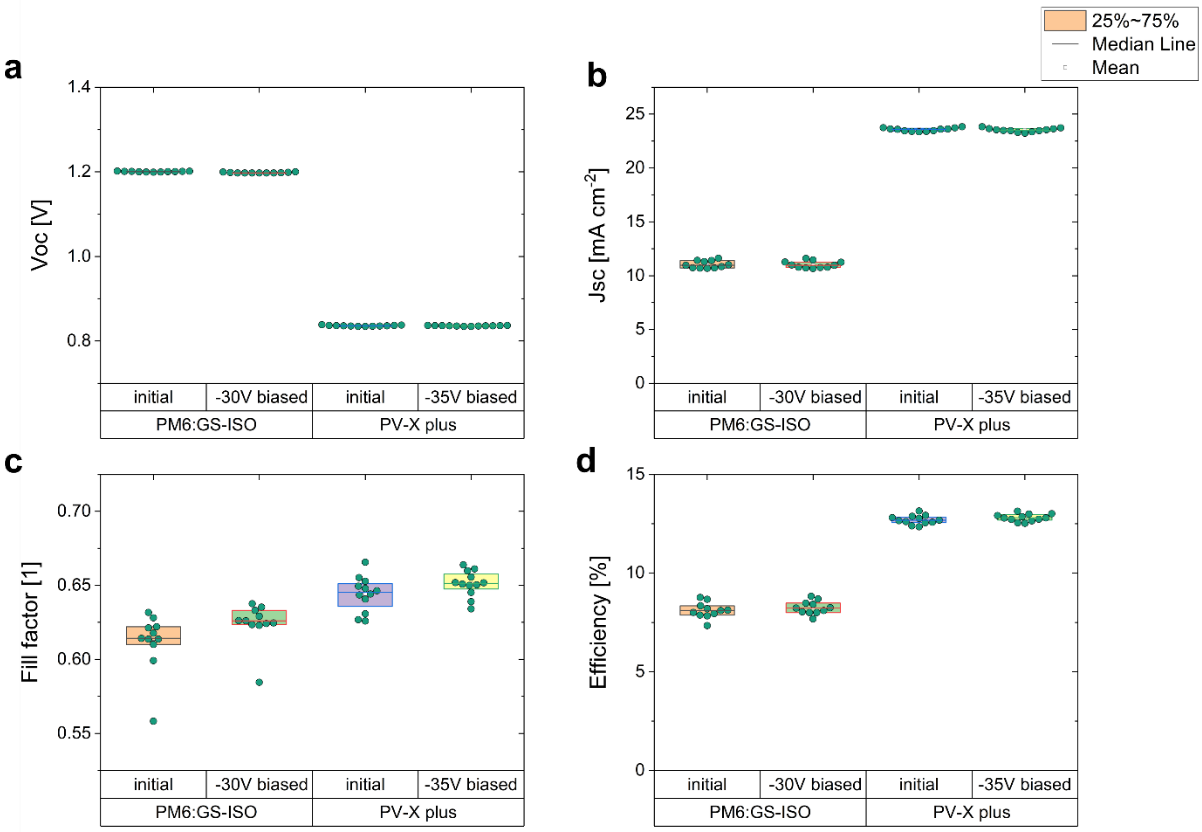

Supplementary Fig. 15 | Impact of large reverse bias on the performance of solar cells. **a**, Open circuit voltage (V_{OC}). **b**, Short circuit current density (J_{SC}). **c**, Fill factor. **d**, Efficiency. Measured under AM1.5G illumination.

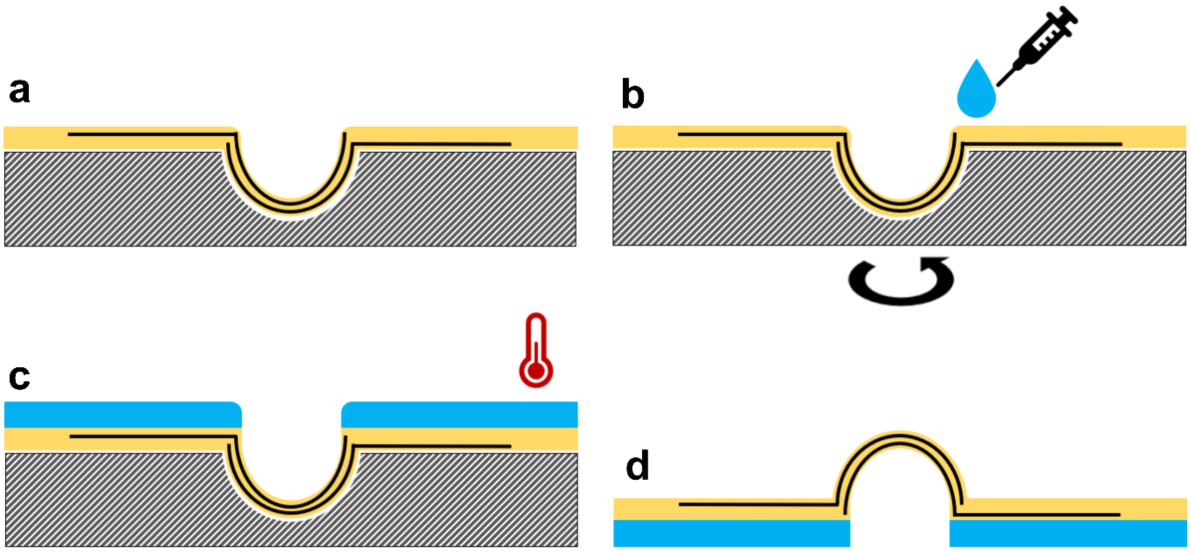

Supplementary Fig. 16 | Fabrication process of dielectric elastomer suction cup. a, The flat DEA is placed onto a mold with a paraboloid cavity. **b**, A silicone compound is applied onto the flat DEA as a backbone material with an offset from the center and starting from the edge of the paraboloid while the mold is spinning. **c**, Curing the backbone material at 80 °C. **d**, Removing the actuator from the mold.

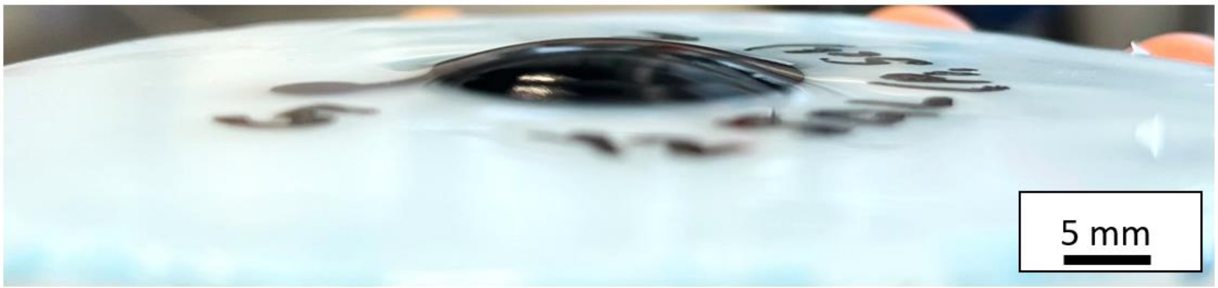

Supplementary Fig. 17 | Photograph of a fully soft dielectric elastomer suction cup.

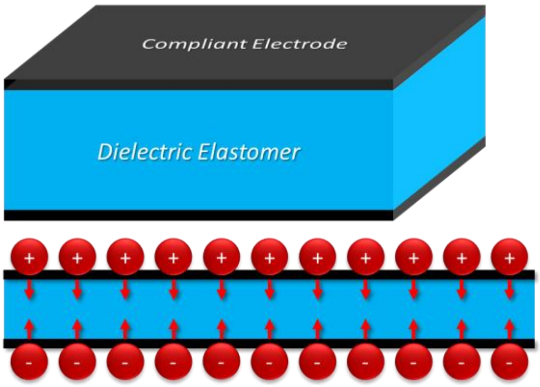

Supplementary Fig. 18 | Deformation of a dielectric elastomer actuator under voltage. As a result of Maxwell stress, the actuator gets thinner and wider.

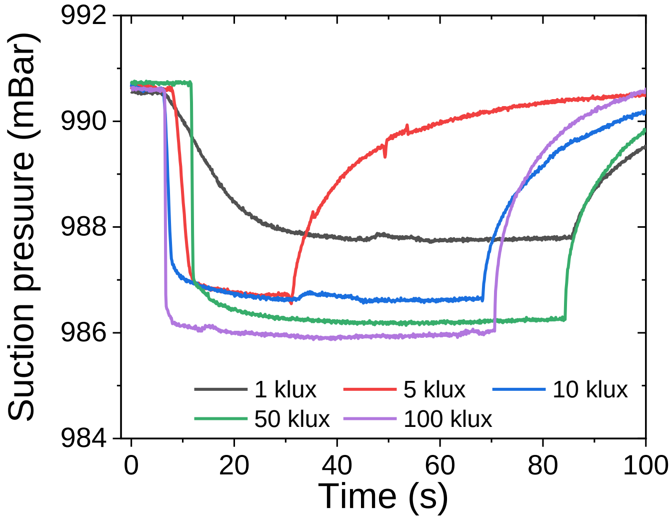

Supplementary Fig. 19 | Transient suction pressure generated by the suction cup. The suction cup was powered by a PM6:GS-ISO high-voltage photovoltaic mini-module under different illuminances.

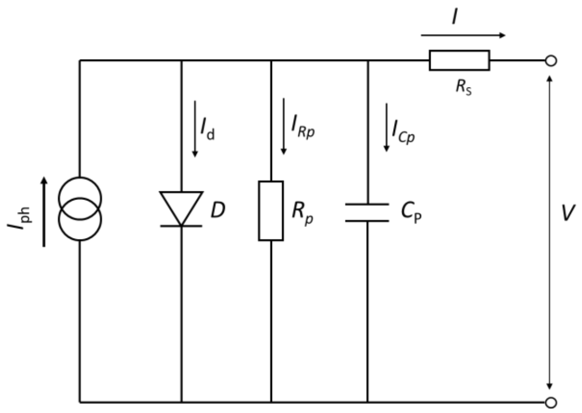

Supplementary Fig. 20 | Equivalent circuit of solar cell. A parallel capacitance (C_p) is included for transient analysis.

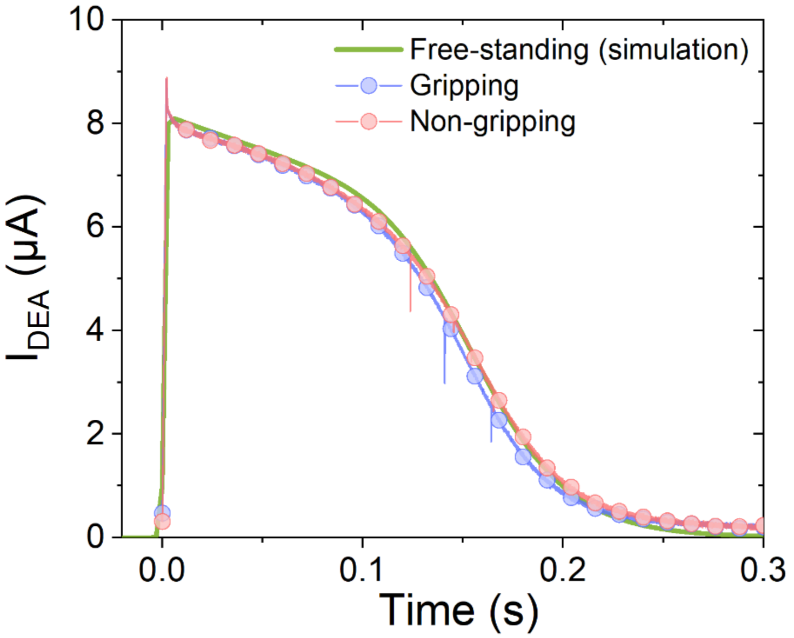

Supplementary Fig. 21 | Charging current of the dielectric elastomer actuator suction cup.

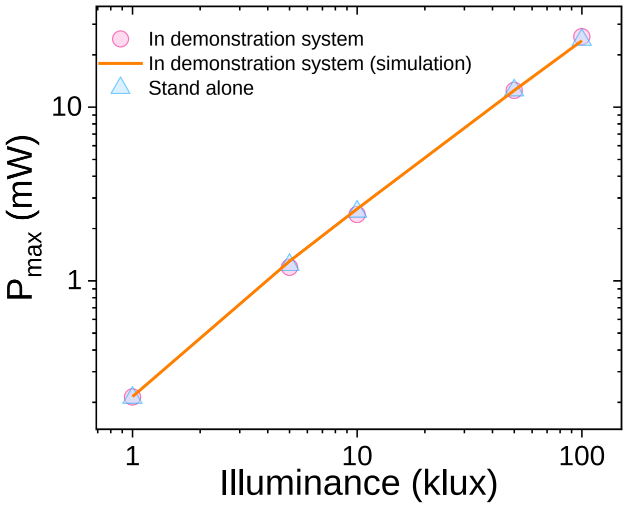

Supplementary Fig. 22 | Maximum power (P_{max}) of the PM6:GS-ISO photovoltaic mini-module. The triangle symbols were extracted from current-voltage measurements of the photovoltaic mini-module not connected to the DEA suction cup.

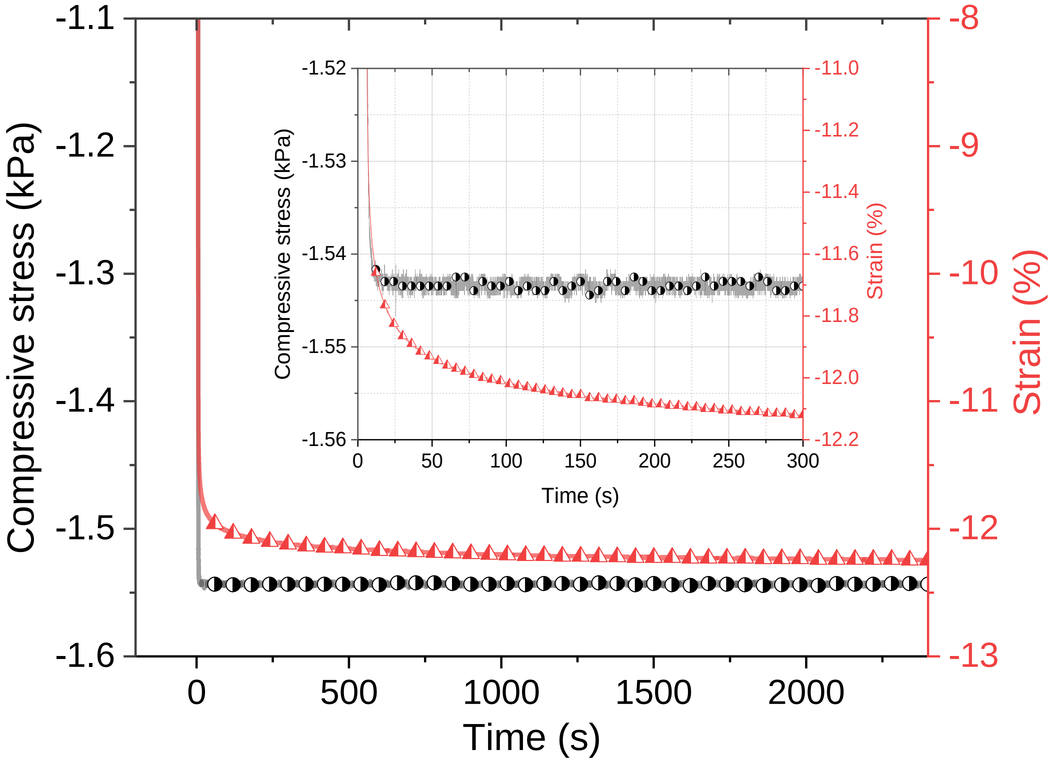

Supplementary Fig. 23 | Viscoelastic behavior of the dielectric elastomer actuator.

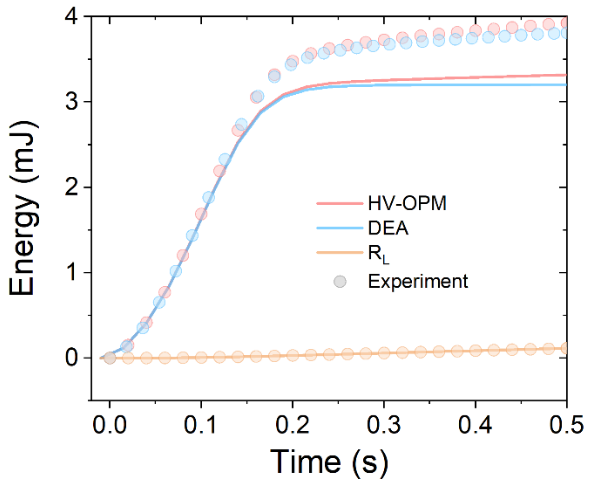

Supplementary Fig. 24 | Details energy analysis. The energy provided by the high-voltage photovoltaic mini-module (HV-OPM) can be divided into the part consumed by the DEA suction cup and the part dissipated in the RL.

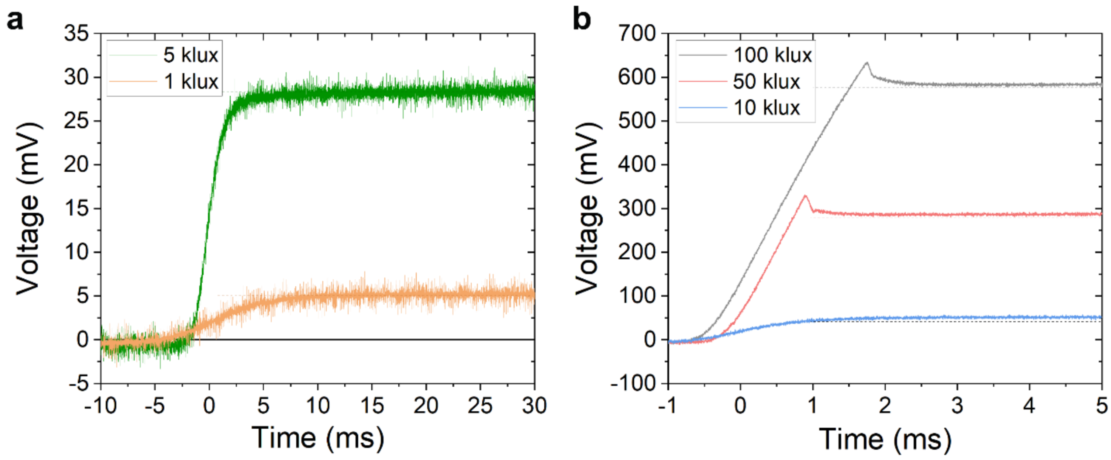

Supplementary Fig. 25 | Turning on feature of the warm white LED lamp. **a**,**b**, Measured photodiode response when turning on LED lamp for illuminances of (**a**) 1-5 klux and (**b**) 10-100 klux. The LED light was filtered with a neutral density filter (optical density=2.3).

Supplementary Tables

Supplementary Table 1. Photovoltaic performance of high-voltage photovoltaic mini-modules.

Sample	Illum. (klux)	P_{in} (mW/cm^2)	V_{OC} (V)	I_{SC} (μA)	*FF*	P_{max} (mW)	PCE^a (%)	PCE^b (%)
PM6:GS-ISO (device 1)	1	0.305	4783	0.083	0.570	0.23	5.7	4.9
	5	1.526	5073	0.402	0.588	1.20	6.0	5.2
	10	3.051	5197	0.822	0.600	2.56	6.4	5.6
	50	15.26	5447	4.045	0.587	12.9	6.4	5.6
	100	30.51	5534	8.118	0.570	25.6	6.4	5.6
PM6:GS-ISO (device 2)	1	0.305	4562	0.076	0.519	0.18	4.5	3.9
	5	1.526	5022	0.395	0.585	1.16	5.8	5.0
	10	3.051	5141	0.783	0.601	2.42	6.0	5.3
	50	15.26	5404	3.882	0.581	12.2	6.1	5.3
	100	30.51	5522	7.698	0.562	23.9	5.9	5.2
PM6:GS-ISO (device 3)	1	0.305	3424	0.067	0.391	0.09	2.2	2.0
	5	1.526	4930	0.355	0.498	0.87	4.3	3.8
	10	3.051	5091	0.710	0.564	2.04	5.1	4.4
	50	15.26	5402	3.514	0.568	10.8	5.4	4.7
	100	30.51	5485	6.974	0.548	21.0	5.2	4.6
PV-X plus (device 4)	1	0.305	2828	0.290	0.683	0.56	13.9	12.2
	5	1.526	3062	1.453	0.727	3.23	16.1	14.1
	10	3.051	3182	2.899	0.722	6.66	16.6	14.5
	50	15.26	3414	14.28	0.681	33.2	16.5	14.4
	100	30.51	3525	28.08	0.641	63.5	15.8	13.8
PV-X plus (device 5)	1	0.305	3387	0.293	0.710	0.71	17.6	15.3
	5	1.526	3626	1.476	0.762	4.08	20.3	17.7
	10	3.051	3717	2.961	0.760	8.37	20.8	18.2
	50	15.26	3902	14.73	0.707	40.7	20.2	17.7
	100	30.51	3970	29.23	0.657	76.2	19.0	16.6
PV-X plus (device 6)	1	0.305	3179	0.299	0.524	0.50	12.4	10.8
	5	1.526	3464	1.497	0.715	3.71	18.5	16.1
	10	3.051	3566	2.997	0.743	7.94	19.8	17.3
	50	15.26	3756	14.87	0.732	40.9	20.3	17.8
	100	30.51	3825	29.53	0.694	78.3	19.5	17.0

Note: PCE^a is calculated with respect to the active area (determined by multiplying the active area of a sub-cell with 5024, resulting 13.17 cm^2), PCE^b with respect to the full area (determined by multiplying the full area of a sub-cell with 5024, resulting 15.07 cm^2).

Supplementary Table 2. Photovoltaic performance of single solar cells

Absorber material	Illuminance (klux)	J_{SC} (μA/cm^2)	V_{OC} (V)	*FF*	*PCE* (%)
PV-X plus	1	114.7	0.719	0.771	20.8
	5	560.4	0.762	0.796	22.3
	10	1121.5	0.779	0.791	22.6
	50	5616.1	0.811	0.761	22.7
	100	11249.7	0.82	0.730	22.1
PM6:GS-ISO	1	83.0	1.07	0.700	20.4
	5	424.0	1.12	0.727	22.7
	10	860.1	1.14	0.720	23.2
	50	4278.2	1.18	0.682	22.5
	100	8548.0	1.19	0.658	21.9

Supplementary Table 3. Irreversible breakdown voltage (V_{br}), current density (J_{br}) and power density (P_{br}) of solar cells.

**Samples**	V_{br} (V)	J_{br} (mA/cm^2)	P_{br} (mW/cm^2)
**PV-X plus-cell 1**	40.7	101.6	4135.12
**PV-X plus-cell 2**	42.2	124.5	5253.9
**PV-X plus-cell 3**	41.9	114.1	4780.79
**PV-X plus-cell 4**	42.0	116.8	4905.6
**PV-X plus average**	41.7 ± 0.68	114.3 ± 9.51	4768.8 ± 467.5
**PM6:GS-ISO-cell 1**	35.7	33.1	1181.67
**PM6:GS-ISO-cell 2**	36.0	26.2	943.2
**PM6:GS-ISO-cell 3**	36.3	33	1197.9
**PM6:GS-ISO-cell 4**	36.6	27.7	1013.82
**PM6:GS-ISO average**	36.2 ± 0.39	30.0 ± 3.57	1084.1 ± 125.5

Supplementary Table 4. The parameters of PM6:GS-ISO mini-module used for modeling.

**Illuminance (klux)**	$\boldsymbol{R_{P,m}}$ **(Ω)**	$\boldsymbol{I_{ph}}$ **(A)**	$\boldsymbol{I_o}$ **(A)**	$\boldsymbol{R_{S,m}}$ **(Ω)**	$\boldsymbol{n_{id,m}}$
**1**	2.14E+11	7.96E-08	1.02E-14	2.96E+01	1.12E+04
**5**	5.05E+10	4.22E-07	1.09E-13	5.95E+01	1.24E+04
**10**	2.63E+10	8.44E-07	6.05E-13	2.74E+01	1.37E+04
**50**	5.55E+09	4.20E-06	9.94E-11	5.60E+01	1.93E+04
**100**	2.89E+09	8.28E-06	1.05E-09	3.03E+01	2.38E+04

Supplementary Table 5. The parameters of the DEA suction cup used for modeling.

**Name**	**Description**	**Value**
$C_{DAE,0}$	DEA capacitance at 0V	208 pF
z_0	Initial DEA layer thickness	550 µm
ε_r	Relative dielectric permittivity of the DEA material	3.9
Y	Young’s modulus of the DEA material	27 kPa
$R_{S,DEA}$	series resistance of the DEA	14 MΩ
$R_{P,DEA}$	parallel resistance of the DEA	1.0 TΩ

References

1. Li, K. *et al.* Breakdown mechanisms and reverse current-voltage characteristics of organic bulk heterojunction solar cells and photodetectors. *Journal of Applied Physics* **115**; 10.1063/1.4883501 (2014).
2. Szaniawski, P., Lindahl, J., Törndahl, T., Zimmermann, U. & Edoff, M. Light-enhanced reverse breakdown in Cu(In,Ga)Se2 solar cells. *Thin Solid Films* **535,** 326–330; 10.1016/j.tsf.2012.09.022 (2013).
3. Jamali, A., Mishra, D. B., Goldschmidtboeing, F. & Woias, P. Soft octopus-inspired suction cups using dielectric elastomer actuators with sensing capabilities. *Bioinspiration & biomimetics* **19**; 10.1088/1748-3190/ad3266 (2024).
4. Tang, C. *et al.* A Review on High-Frequency Dielectric Elastomer Actuators: Materials, Dynamics, and Applications. *Advanced Intelligent Systems*; 10.1002/aisy.202300047 (2023).
5. Madsen, F. B., Daugaard, A. E., Hvilsted, S. & Skov, A. L. The Current State of Silicone-Based Dielectric Elastomer Transducers. *Macromolecular rapid communications* **37,** 378–413; 10.1002/marc.201500576 (2016).
