# Supplementary material for: Organic photovoltaic mini-module providing more than 5000 V for energy autonomy of dielectric elastomer actuators
Source: Nat Commun. 2025 Feb 28;16:2048. doi: 10.1038/s41467-025-57226-6 (PMC11871061; doi:10.1038/s41467-025-57226-6)
Supplement: Supplementary file 2 — Description of Additional Supplementary Information [file 41467_2025_57226_MOESM2_ESM.docx]

**Supplementary Video 1.**

Gripping Demonstration of a DEA Suction Cup Powered by a High-Voltage Solar Mini-Module

This video demonstrates the gripping mechanism of a DEA-based suction cup powered by a high-voltage solar mini-module. The experiment is conducted under ambient conditions.
