# Supplementary material for: Organic photovoltaic mini-module providing more than 5000 V for energy autonomy of dielectric elastomer actuators
Source: Nat Commun. 2025 Feb 28;16:2048. doi: 10.1038/s41467-025-57226-6 (PMC11871061; doi:10.1038/s41467-025-57226-6)
Supplement: Supplementary file 4 — Reporting Summary [file 41467_2025_57226_MOESM4_ESM.pdf]

nature portfolio

Corresponding author(s): Uli Würfel

Solar Cells Reporting Summary

Nature Portfolio wishes to improve the reproducibility of the work that we publish. This form is intended for publication with all accepted papers reporting the characterization of photovoltaic devices and provides structure for consistency and transparency in reporting. Some list items might not apply to an individual manuscript, but all fields must be completed for clarity.

For further information on Nature Research policies, including our data availability policy, see Authors & Referees.

• Experimental design

Please check the following details are reported in the manuscript, and provide a brief description or explanation where applicable.

1. Dimensions

Item	Yes/No	Description
Area of the tested solar cells	[x] Yes [ ] No	Active areas are 0.27 mm^2 for PV-X plus and 0.24 mm^2 for PM6:GS-ISO single cells / *Explain why this information is not reported/not relevant.*
Method used to determine the device area	[x] Yes [ ] No	By measuring cell length and width under microscope. Can be found in method part and supplementary information. / *Explain why this information is not reported/not relevant.*

2. Current-voltage characterization

Item	Yes/No	Description
Current density-voltage (J-V) plots in both forward and backward direction	[ ] Yes [x] No	Organic solar cells do not show hysteresis.
Voltage scan conditions	[x] Yes [ ] No	For the photovoltaic modules, scan direction is forward, with a step of 30 V, speed of 40 V/s, dwell time of 0.15 s. / *Explain why this information is not reported/not relevant.*
Test environment	[x] Yes [ ] No	The modules and single cells are encapsulated first and then tested in ambient air at room temperature. / *Explain why this information is not reported/not relevant.*
Protocol for preconditioning of the device before its characterization	[x] Yes [ ] No	Encapsulated before the measurement, no further pre-conditioning / *Explain why this information is not reported/not relevant.*
Stability of the J-V characteristic	[ ] Yes [x] No	*Provide a description of the method used. The stability of the J-V characteristic can be verified with time evolution of the maximum power point or with the photocurrent at maximum power point; see ref. 5 for details.* / The highlight of this work is the very high voltage but not the efficiency.

3. Hysteresis or any other unusual behaviour

Item	Yes/No	Description
Description of the unusual behaviour observed during the characterization	[ ] Yes [x] No	*Provide a description of hysteresis or any other unusual behaviour observed during the characterization.* / Unusual behaviour was not observed.
Related experimental data	[ ] Yes [x] No	*Provide a description of the related experimental data.* / Unusual behaviour was not observed.

4. Efficiency

Item	Yes/No	Description
External quantum efficiency (EQE) or incident photons to current efficiency (IPCE)	[ ] Yes [x] No	*Provide a description of the technique used.* / The highlight of this work is the very high voltage but not the efficiency.
A comparison between the integrated response under the standard reference spectrum and the response measure under the simulator	[ ] Yes [x] No	*State where this information can be found in the text.* / The highlight of this work is the very high voltage but not the efficiency.

For tandem solar cells, the bias illumination and bias voltage used for each subcell	☐ Yes<br>☒ No	*Provide a description of the measurement conditions.*<br>We do not report tandem solar cells.

5. Calibration

Light source and reference cell or sensor used for the characterization	☒ Yes<br>☐ No	Warm white LED lamp (LTAS-100/1 from Brandmaier), Reference cell is RS-ID-4<br>*Explain why this information is not reported/not relevant.*
Confirmation that the reference cell was calibrated and certified	☒ Yes<br>☐ No	Calibrated at CalLab, Fraunhofer ISE<br>*Explain why this information is not reported/not relevant.*
Calculation of spectral mismatch between the reference cell and the devices under test	☐ Yes<br>☒ No	*Provide a value of the spectral mismatch and/or a description of how it has been taken into account in the measurements.*<br>We are not reporting standard AM1.5G efficiency.

6. Mask/aperture

Size of the mask/aperture used during testing	☐ Yes<br>☒ No	*Report the size of the mask/aperture.*<br>The small areas were laser patterned and determined precisely by microscope.
Variation of the measured short-circuit current density with the mask/aperture area	☐ Yes<br>☒ No	*Report the difference in the short-circuit current density values measured with the mask and aperture area.*<br>No aperture was used.

7. Performance certification

Identity of the independent certification laboratory that confirmed the photovoltaic performance	☐ Yes<br>☒ No	*Identify the independent certification laboratory.*<br>The highlight of this work is the very high voltage but not the efficiency.
A copy of any certificate(s)	☐ Yes<br>☒ No	*Certificate copies should be provided in the Supplementary information. Please state the supplementary item number.*<br>The highlight of this work is the very high voltage but not the efficiency.

8. Statistics

Number of solar cells tested	☒ Yes<br>☐ No	For each absorbers, one single solar cell (6 on one substrate) and 3 mini-modules were tested.<br>*Explain why this information is not reported/not relevant.*
Statistical analysis of the device performance	☒ Yes<br>☐ No	In the main text and in Fig. 3<br>*Explain why this information is not reported/not relevant.*

9. Long-term stability analysis

Type of analysis, bias conditions and environmental conditions	☒ Yes<br>☐ No	A cool white LED lamp with an illuminance of 50 klux was used. Modules were encapsulated with DELO®KATIOBOND®LP655. Aging was done in ambient air without control of temperature and humidity. The module were kept under open circuit or short circuit conditions.<br>*Explain why this information is not reported/not relevant.*
